# Characterization and functional analysis of hypoxia-inducible factor HIF1α and its inhibitor HIF1αn in tilapia

**DOI:** 10.1371/journal.pone.0173478

**Published:** 2017-03-09

**Authors:** Hong Lian Li, Xiao Hui Gu, Bi Jun Li, Xiao Chen, Hao Ran Lin, Jun Hong Xia

**Affiliations:** State Key Laboratory of Biocontrol, Institute of Aquatic Economic Animals and Guangdong Provincial Key Laboratory for Aquatic Economic Animals, College of Life Sciences, Sun Yat-Sen University, Guangzhou, PR China; University of Nebraska Medical Center, UNITED STATES

## Abstract

Hypoxia is a major cause of fish morbidity and mortality in the aquatic environment. Hypoxia-inducible factors are very important modulators in the transcriptional response to hypoxic stress. In this study, we characterized and conducted functional analysis of hypoxia-inducible factor HIF1α and its inhibitor HIF1αn in Nile tilapia (*Oreochromis niloticus*). By cloning and Sanger sequencing, we obtained the full length cDNA sequences for HIF1α (2686bp) and HIF1αn (1308bp), respectively. The CDS of HIF1α includes 15 exons encoding 768 amino acid residues and the CDS of HIF1αn contains 8 exons encoding 354 amino acid residues. The complete CDS sequences of HIF1α and HIF1αn cloned from tilapia shared very high homology with known genes from other fishes. HIF1α show differentiated expression in different tissues (brain, heart, gill, spleen, liver) and at different hypoxia exposure times (6h, 12h, 24h). HIF1αn expression level under hypoxia is generally increased (6h, 12h, 24h) and shows extremely highly upregulation in brain tissue under hypoxia. A functional determination site analysis in the protein sequences between fish and land animals identified 21 amino acid sites in HIF1α and 2 sites in HIF1αn as significantly associated sites (α = 0.05). Phylogenetic tree-based positive selection analysis suggested 22 sites in HIF1α as positively selected sites with a *p*-value of at least 95% for fish lineages compared to the land animals. Our study could be important for clarifying the mechanism of fish adaptation to aquatic hypoxia environment.

## Introduction

The hypoxia signaling pathway is an evolutionarily conserved signaling pathway present in many kinds of animals which is crucial for oxygen homeostasis maintenance [1]. Hypoxia-inducible factors (HIFs) are the key modulators in the transcriptional response to hypoxic stress [2]. HIFs are heterodimers consisting of an O_2_-regulated α subunit and a constitutively expressed β subunit [3]. HIF α subunits (HIFαs) and HIF β subunits (HIFβ) are members of the bHLH/PAS protein family, containing one N-terminal basic-helix-loop-helix (bHLH) domain which mediates DNA binding and two Per-ARNT-Sim (PAS) domains which work in dimerization [4]. HIF heterodimers recognize and bind to hypoxia response elements (HREs) in the genome, and then activate or inhibit the expression of subsequent genes [5]. Transcriptional responses to hypoxia are primarily mediated by HIFαs [6]. In mammals, there exists three isoforms of HIFα, i.e., HIF1α, HIF2α and HIF3α. Of which, HIF1α and HIF2α are structurally conserved and best studied, while HIF3α is the most structurally distant factor among HIFs [7]. HIF1α is expressed ubiquitously in all cells, whereas HIF2α and HIF3α are selectively expressed in certain tissues and cells [8]. HIFα are directly or indirectly regulated by many factors, such as HIF1αn (HIF1α inhibitor, also known as FIH1), PHD1–3 (also known as EGLN1–3), microRNAs, sirtuins [9], NF-κB [10], SUMO-1 [11]. HIF1αn is a HIF hydroxylase, functioning as an oxygen sensor. HIF1αn could hydroxylate HIFα by catalyzing HIFα hydroxylation of asparaginyl residue and leads to reduced interaction of HIF with the transcriptional co-activator proteins CBP/p300 [12].

Oxygen concentration in water varies more significantly over time and space than in terrestrial environment, thus hypoxia is a common phenomenon for lives living in water [13]. Hypoxia induces many physiological and biochemical changes of fishes. For example, increasing blood oxygen-carrying capacity, anaerobic metabolism proportion [14], food intake and growth [15, 16], blood parameters changes [17, 18], decreasing reproductive capacity and embryonic development [19–21], disrupting sex hormone metabolism and altering the sexual differentiation [22] and cardiorespiratory function [23, 24]. Some important factors related to hypoxia adaptation and the hypoxia signaling pathway has also been identified in fish. Previous studies about hypoxia in fish species have focused on transcriptome responses to hypoxia (mainly HIF pathway), because HIFs are the main regulators of many downstream genes involved in many kinds of metabolic pathway [1, 13, 25–27] and hypoxia adaptation [13, 28–30]. To date, HIF1α sequences have been reported in several fish species, such as rainbow trout [31], eelpout [32], zebrafish [33], crucian carp [34], grass carp [35], Atlantic croaker [36]. The structures and functions of fish HIFs are similar to those of mammals [1]. The studies about HIF1αn are few. HIF1αn may interact with Mib E3 Ubiquitin ligase and negatively effects vascular development by attenuating VEGF-A signaling activity in zebrafish [37]. The expression of HIF1αn mRNA after hypoxia treatment was complex, and in general longer period of hypoxia induced up-regulation of HIF1αn in most tissues which may represent a feedback mechanism [13].

Tilapia is a hypoxia-tolerant fish known as its high adaptability and fast growth performance [38]. It can naturally deal with abrupt fluctuations in oxygen availability without having to face the danger of excessive oxidative stress [38]. So, it represents an excellent model for hypoxia research [38]. Currently, little hypoxia related molecular data in this fish is available. In tilapia, acute hypoxia triggered an overall down-regulation of the immune system [39] and moderate hypoxia negatively affects antibody production, ultimately affecting vaccine efficacy [40]. The depression of whole animal oxygen consumption rate, heart rate and cardiac output were found contribute to hypoxic survival [41]. Norepinephrine may be responsible for the reduced plasma free fatty acid concentration under hypoxia through inhibition of lipolysis in adipose tissue [42]. Hypoxic depression of plasma free fatty acid in tilapia does not play a key role in cardiac hypoxia tolerance [43]. The distribution and orientation of gill O_2_ chemoreceptors plays a role in cardiorespiratory responses to graded hypoxia [44]. Recent study shows miRNA miR-204 could regulate VEGF expression in tilapia [38].

Teleost fish live in the aquatic environment [29]. Hypoxia is a major cause of fish morbidity and mortality [29]. Many fishes have evolved the ability to survive extended periods of exposure to hypoxia [45]. Learning about the mechanism of gene regulation under hypoxia stress in fish is one of the very important fields in fish biology. In this study, we investigated the structures, expression characteristics of the gene HIF1α and its inhibitor HIF1αn in an economic important fish species, Nile tilapia, *Oreochromis niloticus*, under normoxic and hypoxic conditions and evaluated positive selections of HIF1α and HIF1αn related to aquatic environment adaptation. Our study could contribute to understand the mechanism of hypoxia adaptation in fish.

## Materials and methods

### Fish and experiment condition

Nile tilapia samples used in expression experiment are reproduced and cultured in an indoor water recirculation system in the life sciences school of Sun Yet-sen University. Thirty six tilapia at 90 day post hatch were randomly divided into two tanks (125L). Each tank contains 18 fishes and kept at the normal dissolved O_2_ (DO) concentration (7.52±0.33 mg/l) by continuous incharging the air into the water. After one day acclimation, the DO concentration in one tank were adjusted to hypoxia condition (0.70±0.21 mg/l) by nitrogen aeration, the DO concentration in the other tank was kept at the normal O_2_ concentration (7.52±0.33 mg/l) and used as control. Tissue samples (heart, liver, brain, gill and spleen) from 5 fishes from each tank were collected at 6h, 12h and 24h post hypoxia treatment, respectively. The Animal Care and Use Committee the life sciences school of Sun Yet-sen University has approved this research.

One GIFT tilapia population crossed from hundreds of individuals was bought from a local fish hatchery of Guangdong province, China. The populations consisting of ~ 300 individuals at ~ 20 dph was transferred to one one-ton tank with circulating water in the fish facility of Life Sciences School, Sun Yat-sen university, Guangzhou, China. Fish were fed with tilapia pellets twice a day. The water temperature was 25–28°C and the DO maintained at >5 mg O2/L during the experiments. At ~ dph 120, the water DO in the tank was quickly decreased to an acute hypoxia (~1 mg O2/L) by pumping nitrogen into water through a nitrogen gas cylinder. We recorded the time point that a fish falls unconscious under hypoxia stress. Then, the surviving time for each fish was calculated by using the time point that the fish show unconscious minus the time point that recorded for the first unconscious fish. We also collected the fin sample for each fish in absolute ethanol for DNA extraction.

### RNA extraction and cDNA synthesis

RNA was extracted using the TRIzol reagent (Life technologies, USA) according to the manufacturer’s instructions. The quality and quantity of RNA samples were detected by using 1.5% agarose gel electrophoresis and nucleic acid quantitation with NanoDrop 2000 Spectrophotometers (Thermo Scientific, USA). After extraction, 2μg of total RNA from each individual was reversely transcribed into cDNA using RT-PCR kit (Dongsheng biotech, China) according to the manufacturer’s instructions. In brief, a mixture of 2μg DNAse I treated total RNA, 1μl oligo dT primer and RNase-free ddH_2_O in a volume of 13.4μl was kept at 70°C for 5 min and then put in ice bath for 5 min. 6.6μl of mixture (including 4μl of 5×first-strand buffer, 1μl dNTPs, 0.6μl RNasin and 1μl M-MLV) were added into the tube and kept at 42°C for 60 min. A final step keeps at 70°C for 5 min to end the reaction. The thermal cycles were conducted in a PCR machine (BIO-RAD, USA). The obtained cDNA was kept at -20°Cuntil use.

### Sequencing, genotyping and gene structure prediction

Primers were designed and used to clone HIF1α and HIF1αn sequences according to their homolog sequences in NCBI database. Both the forward and reverse primers matched to the outlier region of the CDS, thus the full-length CDS sequences were covered. The primers were listed in the S1 Table. A PCR procedure was carried out with a PCR kit (DSMix; Dongsheng biotech, China) according to the manufacturer’s instructions. After purification by a column-based method, the PCR products were sent for Sanger sequencing directly. The SNP calls for the 192 individuals from the hypoxia treated population with extreme traits were manually conducted.

The resulted sequences were blasted against the nt database in NCBI to locate the positions and sizes of introns and exons in HIF1α and HIF1αn genes. The gene structures of HIF1α and HIF1αn were drawn with the on line tool Gene Structure Display Server 2.0 (GSDS2.0) (http://gsds.cbi.pku.edu.cn/) by inputting their location information of UTRs, introns and exons. The conserved domains were analyzed by using the online tool SMART (http://smart.embl-heidelberg.de/). The GenBank accession numbers for HIF1α and HIF1αn are KY415998 and KY415999, respectively.

### Real-time quantitative PCR (qPCR)

The relative expression levels of HIF1α and HIF1αn mRNAs in different tissues and at different exposure times under hypoxia were investigated by qPCR. The primers used were listed in the S1 Table. EF1α was used as internal reference. The qPCR was performed using SYBR Green qPCR mix (Dongsheng biotech, China) on LightCycler480II (Roche, Switzerland). Each sample was performed with three duplicates. cDNA products were diluted 1:10 before used as qPCR templates. The total volume of the reaction system was 20 μl, containing 10 μl 2×SYBR Green mix, 0.4μl forward primer, 0.4 μl reverse primer, 4.2 μl ultrapure water and 5 μl of diluted cDNA. The qPCR program includes 3 min of 95°C pre-incubation, 40 cycles of amplification at 95°C for 15s, 60°C for 15s and 72°C for 20s. The melting curve procedures include 95°C for 15s, 65°C for 15s and a slowly increase to 95°C. The result was analyzed according to the ΔΔCt method [46]. The two-tailed T statistical test in the Microsoft EXCEL software was performed to compare the expression differences between two datasets.

### Sequence data collection, alignment and phylogenetic analysis

In order to inferring the positive selection events during adaptation evolution, the orthologous CDS sequences for HIF1α and HIF1αn genes were downloaded from orthodb7 database (http://cegg.unige.ch/orthodb7/). The CDS sequences from each gene were realigned with the software MUSCLE (Multiple Sequence Comparison by Log-Expectation) [47] with default parameters and the software MACSE (Multiple Alignment of Coding SEquences, v1.01b) [48] with respect to their amino acid translation.

The multiple alignment sequences for each protein coding genes were used to construct a maximum likelihood phylogeny tree by the software codonPhyML (ver 1.00; Fast Maximum Likelihood Phylogeny Estimation under Codon Substitution Models) [49] with parameter setting as -m GY—fmodel F3X4 -t e -f e -w DGAMMA—wclasses 3. The resulting phylogenies were presented with SH-like branch supports >0.5. The phylogenetic trees were drawn with the software MEGA7 [50]. Branches were transformed as cladogram.

### Inferring of positive selection events based on phylogenetic analysis

The significant associated residues in HIF1α and HIF1αn proteins between fishes and land animals were identified by the SigniSite 2.1 Server (www.cbs.dtu.dk/services/SigniSite) [51].

The codeml program from the Phylogenetic Analysis by Maximum Likelihood (PAML) package (available at http://abacus.gene.ucl.ac.uk/software/paml.html) [52] was used to perform a likelihood ratio test of positive selection based on the dN/dS ratio (ω). The branch + site models [53] were used to analyze the CDS data as described in PAML manual [52–53]. Briefly, codeml was run under two models for each gene. Model A defines four classes of sites, where two of the classes have a ω ≤ 1 on all branches while two additional classes have ω > 1 on the lineage of interest (inhabiting water or land environments), but ω ≤ 1 on the other branches of the tree (Sites on the labeled branch allowed to differ; 0 < ω0 <1, ω1 = 1, ω2a ≥1, ω2b ≥1). Model Anull fixes ω = 1 for the latter two classes (0 < ω0 <1, ω1 = 1, ω2a = 1, ω2b = 1) [53]. The comparison of Model A with Model Anull was then carried out as strict tests of positive selection using a likelihood ratio test (LRT) [52]. A P-value was calculated by comparing two times the difference in log likelihood values (LRT = 2*(lnL1-lnL2)) to a chi-squared distribution, with the degrees of freedom equal to the difference in number of parameters between the pair of nested tests [52]. If ω > 1 in the branch model and the LRT is significant (*P* < 0.05), positive selection were inferred [52]. Bayes empirical bayes inference of amino acid sites under positive selection were also carried out by the program codeml [54].

### Association analysis of two genes with hypoxia tolerant traits

We first performed F statistics tests for the differences between genotype/phenotype data in the population (N = 192) in the Microsoft Excel. The program PLINK 1.9 (http://pngu.mgh.harvard.edu/~purcell/plink/index.shtml) was then used to perform an association analysis of genotype/phenotype data using the Wald test (based on t-distribution).

## Results and discussions

### Sequences and structures of the tilapia HIF1α and HIF1αn genes

**Fig 1:**
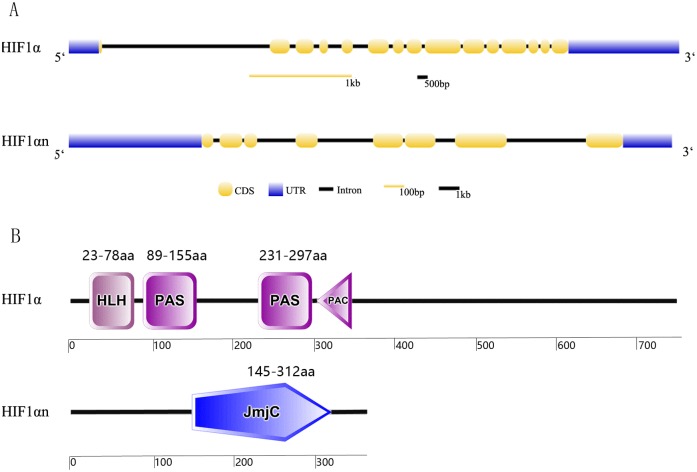
Gene structures (A) and location of conserved domains (B) of the HIF1α and HIF1αn gene and protein cloned from Nile tilapia.

Many fishes have evolved the ability to survive extended periods of exposure to hypoxia [45] by inducing many physiological and biochemical changes in body. HIFs are recognized as key modulators of the transcriptional response to hypoxic stress. There are some researches about hypoxia in tilapia, but little is concerning on HIF1α and HIF1αn. By cloning and Sanger sequencing, we obtained the full length cDNA sequences for HIF1α (2686bp) and HIF1αn (1308bp), respectively, and confirmed the accuracy by blasting their sequences against the tilapia genomes (http://www.ncbi.nlm.nih.gov/genome/197). We found the two cloned genes contain highly similar sequences to the predicted HIF1α (NC_022217.1, location: 1825272..1840183) and HIF1αn (NC_022211.1, location: 20608233..20619993) in tilapia genome. The CDS sequences of HIF1α and HIF1αn we cloned both have 99% similarity with corresponding mRNA sequences (HIF1α Sequence ID: XM_005477039 and HIF1αn Sequence ID: XM_003452002) in NCBI database. The total gene length including the intron, exon and UTRs is 14,912bp and 11,761bp for HIF1α and HIF1αn, respectively. HIF1α cDNA included 15 exons which encoding 768 amino acid residues. HIF1αn cDNA contains 8 exons which encoding 354 amino acid residues. Of the fourteen introns identified in the HIF1α, the first one was the longest, while HIF1αn had only seven introns in which the last one was the longest. The gene structures of HIF1α and HIF1αn were showed in Fig 1A.

We translated the HIF1αn and HIF1α CDS sequences and analyzed the conserved domains in the protein sequences using the on line tool SMART [55]. In tilapia HIF1α protein, we found the common conserved HLH domain and two PAS domains. HIF functions in the form of heterodimers [4]. HLH domain plays roles in DNA binding with HIF and PAS domains act in HIF1α and HIF1β dimerization [4]. In the HIF1αn protein, we found a conserved JmjC domain. Most JmjC proteins have histone demethylation function but some others have functions independent of histone demethylation, such as HIF1αn function as a protein hydroxylase [56]. The high conservation of these two genes in many fish species illustrates their important functions during evolution. The order and location of these conserved domains in the two protein sequences were showed in Fig 1B.

### Relative spatial and temporal expression of HIF1α and HIF1αn under normoxic and hypoxia conditions

**Fig 2:**
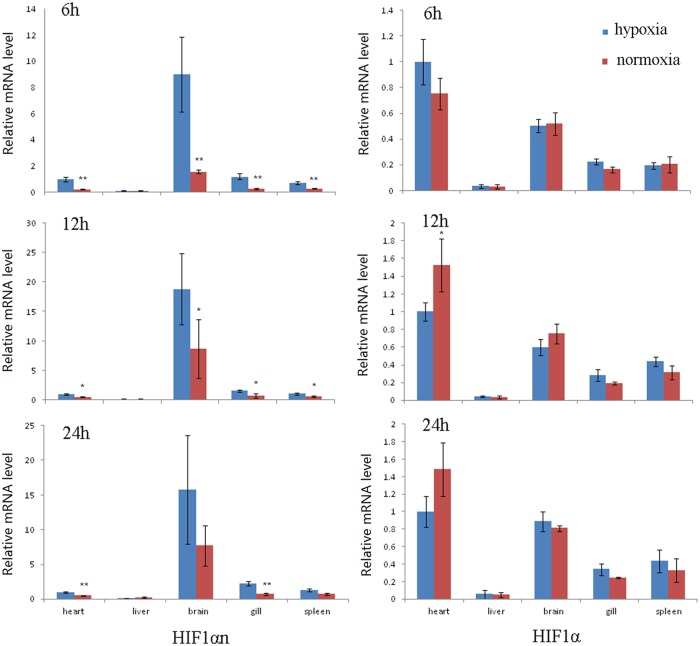
The relative expression of HIF1αn and HIF1α post 6h, 12h and 24h hypoxia treatment in tilapia heart, liver, brain, gill and spleen tissues. EF1α was used as internal control, and all values are normalized to value of heart under hypoxia. The bars indicate mean value of three fish. Significant level: ‘*’, *p*<0.05 and ‘**’,<0.01.

The tilapia HIF1α has maximum expression level in heart, then in brain, and minimum expression level in liver at all three time points as indicated by qRT-PCR (Fig 2). HIF1α expression in heart under hypoxia is slightly higher than that under normoxic condition at 6h, but is significantly higher in normoxic than hypoxia conditions at 12h (*P* < 0.05) and slightly higher at 24h (*P* > 0.05). In gill tissue, HIF1α expression under hypoxia was more than those under normoxic at all-time points, but the differences were statistically insignificant. HIF1α expression in spleen was slightly increased in hypoxia compared with normoxic condition at 12h and 24h (*P* > 0.05), except at 6h which has similar expression level in hypoxia and normoxic. Previous studies in many mammals and some fish species indicated up-regulation of HIF1α under hypoxia condition. But this up-regulation is tissue, fish species and hypoxia condition dependent. For example, in *Perca fluviatilis*, a hypoxia-sensitive fresh water species, an up-regulation of HIF1α were indicated in brain and liver, but not in muscle tissue after acute hypoxic treatment, whereas significant changes of HIF1α mRNA levels were detected in muscle, but not in brain and liver after chronic hypoxia exposure [57]. In *Sebastes schlegelii*, different change model for HIF1α expression were found in different tissues and different exposure time under hypoxia [58]. In our study, we found a complex expression model of tilapia HIF1α at different tissues and time.

At all-time points, HIF1αn had the highest expression level in brain and lower in other four tissues, and showed the lowest expression in liver (Fig 2). The HIF1αn expression in the liver at three time points, and in the brain and spleen at 24h were statistically insignificant between control and treated samples (*P* > 0.05). At the remaining tissues and time points, HIF1αn expression level under hypoxia was significantly higher than those under normoxic condition (*P* < 0.05). HIF1αn in some tissues of a highly tolerant to low oxygen condition fish species, channel catfish, was up-regulated under hypoxic conditions [13]. In the gill and muscle of *Macrobrachium nipponense* that exposed to hypoxia for 24h, elevated expression level of HIF1αn was also observed, although no significant differences at 1h and 3h [59]. These studies suggested a general up-regulation of HIF1αn expression under hypoxia condition, illustrating an important function of HIF1αn in fish hypoxia adaptation.

### Phylogenetic relationship and positively selected sites in the HIF1α and HIF1αn

**Fig 3:**
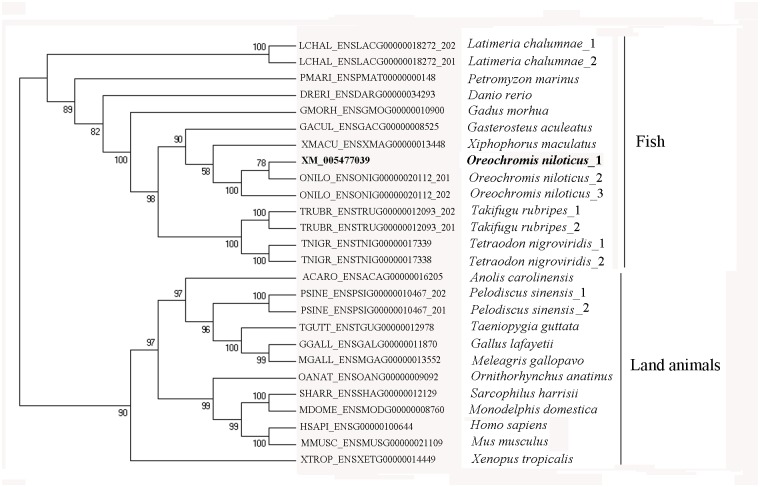
The unrooted phylogenetic trees constructed by using CDS sequences of HIF1α genes showing separating of the fish species from the land animals.

After a comprehensive search in ortholog databases excluding the partial and unfinished cDNA sequences, we picked 25 CDS sequences for HIF1α and 24 CDS sequences for HIF1αn. The HIF1α genes are from 9 fish species and 11 land animals (Fig 3). The HIF1αn gene orthologs originated from 10 fish species and 14 land animals. The dataset of downloaded CDS sequences was aligned to produce a maximum likelihood phylogeny tree for HIF1α (Fig 3) and HIF1αn (Data not provided) by using Fast Maximum Likelihood Phylogeny Estimation under Codon Substitution Models) [49]. The analysis of phylogenetic trees constructed by using CDS sequences of HIF1α and HIF1αn genes strongly supports separating of the fish species from the land animals (Fig 3). The closely related species were clustered to each other. The fish HIF1α were assorted well to fish lineages, and the land animals assorted well to land animal lineages, all with high SH-like branch support values.

**Fig 4:**
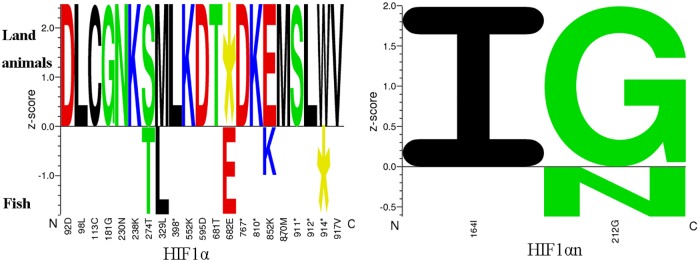
Sequence logo quantifying strength of residues in HIF1αn and HIF1α protein sequences that associated with traits between fishes and land animals. Amino acid residues on the positive y-axis are associated with strong land animal phenotype values and residues on the negative y-axis associated with fish phenotype values. The amino acids are colored according to their chemical properties as follows: Acidic [DE]: red, Basic [HKR]: blue, Hydrophobic [ACFILMPVW]: black and Neutral [GNQSTY]: green.

Teleost fish live in the aquatic environment [29], where the DO is much lower than that in the air. Compared to the land animals, which obtain oxygen from the air, hypoxia is a major factor causing fish morbidity and mortality [29]. Many fishes have evolved the ability to survive extended periods of exposure to hypoxia [45]. To test whether there exist positive sites or not in HIF1α and HIF1αn sequences under adaptive evolution to the aquatic environments, we first identified the functional determination sites in the protein sequences between fish and land animals by using the SigniSite 2.1 Server (www.cbs.dtu.dk/services/SigniSite, Fig 4). The HIF1α alignment has an aligned length of 1056 positions. A total of 3295 tests were performed at a significance level of 5.00% (α = 0.05). After correction for multiple comparisons, using the method 'bonferroni', 21 amino acid sites were identified as significantly associated sites with the fish or land animal phenotype (Fig 4). The HIF1αn alignments have an aligned length of 388 positions. A total of 641 tests were performed at a significance level of 5.00% (α = 0.05). But only two sites were identified as significantly associated amino acid residues. These data may suggest there exist positive selections in the two genes.

The codeml program from the Phylogenetic Analysis by Maximum Likelihood (PAML) package developed by Yang et al [52] can perform a likelihood ratio test of positive selection along specified lineages. We further conducted dN/dS ratio (ω) tests by using the branch + site models from the codeml program [52]. We defined the fish lineages as foreground and the rest as background. The branch + site model detected a ω > 1 in the HIF1α gene, but the LRT is not significant (LRT = 1.4; *P* > 0.05). In the HIF1αn, the ω is < = 1 and the LRT is also not significant (LRT = 0; *P* > 0.05). Therefore, the positive selection for HIF1α and HIF1αn were not inferred based on these data.

Since when more sites are under selection but the pressure is weak, the ‘codeml’ method tends to have trouble identifying them (http://abacus.gene.ucl.ac.uk/software/pamlFAQs.pdf). In the gene HIF1α, for class ω2a, the foreground ω is 1.14854 and have a proportion of 0.15355 and for class ω2b, the foreground ω is 1.14854 and have a proportions of 0.01547. This suggests that there are indeed certain sites under highly variable selective pressures across HIF1α proteins, which is insistent to the results detected with the SigniSite 2.1 Server. We further detected 22 sites as positively selected sites with a *P*-value of at least 95% for foreground lineages Prob(w>1) based on Bayes empirical bayes inference of amino acid sites under positive selection [54]. Some researchers have detected positively selected sites both in the lungfish and amphibian HIF1α and HIF2α in *Sarcopterygii* branch [60]. In another study, researchers detected significant evidence of positive selection in the HIF1α and HIF2β genes in Triplophysa fishes [61]. These researches including our study discovered important functions of HIF1α during hypoxia adaptation. However, in HIF1αn proteins no sites are found under positive selection by using the Bayes empirical bayes inference. Only few researches about HIF1αn were reported. Due to its importance during the hypoxia response in fishes, much more works need be done to discern the selection pressure of the HIF1αn gene in the future.

### Association analysis of genes with hypoxia tolerant traits

To investigate the gene function in the tilapia, we performed an association study between polymorphisms in the genes and the hypoxia tolerance traits in a mass cross population. The phenotypic trait and the fin samples were collected from a study population consisting of 248 individuals. The individuals (N = 56) with medium trait records were removed before genotyping analysis, since it is difficult to order the surviving times for these individuals. Therefore, only 192 individuals with extreme traits were genotyped by sequencing and used in the association study.

We identified one SNP 17835137 G>A (the site position refers to the sequence NCBI Acc no. NC_031983.1) from the fourth intron of the HIF1α gene and four SNPs from the first intron of the HIF1αn gene (Fig 1). The F test indicates there exist significant differences between genotype/phenotypic data at the site SNP 8892953 G>A (refers to the sequence NC_031978.1) in the HIF1αn gene of the population (*P* < 0.01). To further confirm the association, we analyzed the data with the plink program. We found the association of the site SNP 8892953 G>A in the HIF1αn gene was approaching close to significance with the hypoxia tolerance trait (T = -1.856; *P* = 0.07). We observed three genotypes for the SNP marker in the population. The individuals with the SNP genotype A/A (an average surviving time of 15140 seconds under acute hypoxia stress; N = 77) is more tolerant than the other individuals with the genotype A/G (11665 seconds; N = 87) or the genotype G/G (11495 seconds; N = 17). Thus, our studies indicate a potential role for the HIF1αn gene in response to hypoxia stress. Further genotyping of the whole genes in a larger population will help to identify the causative mutations leading to inherited differences in response to hypoxia stress.

### Conclusions

In this study, we investigated the structures, expression characteristics of the gene HIF1α and its inhibitor HIF1αn in Nile tilapia under normoxic and hypoxic conditions. The HIF1α and HIF1αn both showed different spatial expression patterns in five detected tissues. Under normoxic condition, HIF1α and HIF1αn mRNAs were expressed in all tested tissues although expression levels were drastically different. HIF1α expression is complicated in different tissues and at different hypoxia exposure times, while HIF1αn expression level in hypoxia is generally increased. We detected significant evidence of selection signals in HIF1α proteins using different methods. These data may suggest there exist positive selections in the hypoxia-related genes. Our study could contribute to understand the mechanism of hypoxia adaptation in fish.

## Supporting information

S1 Table Primers used for cloning and real-time qPCR of tilapia HIF1α and HIF1αn. (DOCX)

S1 File HIF1α and HIF1αn multiple alignment protein sequences used for identifying the associated residues between fishes and land animals. ACA: *Anolis carolinensis*, PSI:*Pelodiscus sinensis*, TGU:*Taeniopygia guttata*, GAL:*Gallus lafayetii*, MGA:*Meleagris gallopavo*, OAN:*Ornithorhynchus anatinus*, SHA:*Sarcophilus harrisii*, MOD:*Monodelphis domestica*, MUS:*Mus musculus*, XET:*Xenopus tropicalis*, LAC:*Latimeria chalumnae*, PMA:*Petromyzon marinus*, DAR:*Danio rerio*, GMO:*Gadus morhua*, GAC:*Gasterosteus aculeatus*, XMA:*Xiphophorus maculates*, TRU:*Takifugu rubripes*, TNI:*Tetraodon nigroviridis*, LAF:*Loxodonta Africana*, AME:*Ailuropoda melanoleuca*, CPO:*Cavia porcellus*.
(RAR)

S2 File HIF1α multiple alignment cDNA sequences used for positive selection analysis between fishes and land animals. ACA: *Anolis carolinensis*, PSI:*Pelodiscus sinensis*, TGU:*Taeniopygia guttata*, GAL:*Gallus lafayetii*, MGA:*Meleagris gallopavo*, OAN:*Ornithorhynchus anatinus*, SHA:*Sarcophilus harrisii*, MOD:*Monodelphis domestica*, MUS:*Mus musculus*, XET:*Xenopus tropicalis*, LAC:*Latimeria chalumnae*, PMA:*Petromyzon marinus*, DAR:*Danio rerio*, GMO:*Gadus morhua*, GAC:*Gasterosteus aculeatus*, XMA:*Xiphophorus maculates*, TRU:*Takifugu rubripes*, TNI:*Tetraodon nigroviridis*, LAF:*Loxodonta Africana*, AME:*Ailuropoda melanoleuca*, CPO:*Cavia porcellus*.
(RAR)
