# Supplementary material for: Characterization and functional analysis of hypoxia-inducible factor HIF1α and its inhibitor HIF1αn in tilapia
Source: PLoS One. 2017 Mar 9;12(3):e0173478. doi: 10.1371/journal.pone.0173478 (PMC5344420; doi:10.1371/journal.pone.0173478)
Supplement: S1 Table — (DOCX) [file pone.0173478.s001.docx]

**Supplementary Table 1. Primers used for cloning and real-time qPCR of Tilapia** **HIF1α and HIF1αn**

| **Primers** | **Sequence(5’-3’)** |
| --- | --- |
| **Cloning** |  |
| HIF1αF | AAAGTCGACGGAGGATCTGC |
| HIF1αR | CTGGACATCACACGATGCCT |
| HIF1αnF | GCGGAGGTGTATGCTCTCTG |
| HIF1αnR | TGGCACTCGAAGACTGAAGC |
| **qPCR** |  |
| EF1α-qF | GCACGCTCTGCTGGCCTTT |
| EF1α-qR | GCGCTCAATCTTCCATCCC |
| HIF1α-qF | GCACAGTTTGACTTGACTGGAC |
| HIF1α-qR | TTCTTGGAGCCTGTTCTGTGG |
| HIF1αn-qF | TTGGCTATGAGGCTGTCGTG |
| HIF1αn-qR | GGGGCACCTTTGTACCAGA |
